# Foreign Correspondence

**Published:** 1866-02

**Authors:** F. Slayton


### FOREIGN CORRESPONDENCE.

THE following extracts from a letter to the editor, by F. Slayton, D. D. S., of Florence, Italy, may be of some interest to our readers:

DEAR DR. TAFT: — While reading over some old "Registers," and your editorial comments, I made up my mind to have that journal. Father has, for a long time, taken the "Cosmos." I want the Register. Please send it. I have sent you six dollars. * * * Another object in writing was to tell you by what a crowd of savages we are surrounded — all with hammer and saw, and a "Surgeon Dentist" over the door, trying to make artificial dentures. So often, and in so many by places, had the sign, "*Chirurgo Dentista*," met my eye, that I determined, one day, to make a tour of inspection, and send you the result. Happening to be near a certain "*Donati Dentista*," I went up stairs, and asked for the Doctor. It must be that "waiting" is an essential attribute of a dental operation, as I found a little hole, vulgarly called an "ante-waiting saloon," filled with mostly the poorer class of Italians, all waiting to have a tooth "raised." I found an Englishman that wanted a tooth "*stuffed*." Presenting my card, I was, in a second, jerked into another sitting room, and there I met the Doctor, telling him I was an American "fledgeling." He very kindly invited me to take a look at his work-bench, and, also, "with a look of pride," asked me to be seated in the saloon; the said saloon having the most active spiders in the corners, spinning the longest webs that has ever been my fortune to witness. The web extended as follows: commenced at the street door, ran up stairs, passed through the different saloons, into the very sanctum, the operating room, into the patient's pocket, and even had strength enough to draw blood pretty freely from the patient's pocket-book therein to the amount of four gold dollars, for a "swab" of soft silver and plenty of mercury, vulgarly called a "plug." I began to think the Englishman was about right, in coming to get

"stuffed." He only ought to have added "gulled," to make a correct *diagnosis*. Of course the said Donati was a dentist of long standing and of unusual experience, and had used the vulcanite *twenty-two years*; and after having used gold and silver plates for twelve or fifteen years, had renounced all for the carved ivory plate and teeth. His work-shop had every thing but a plane; but I suppose he smoothed down the papillæ with his files. The wood-box served for a dental chair. * * * The Doctor frankly confessed his want of tools, and wanted my case. I could almost forgive his want of necessary tools, so frank was his admission; but the smell of the vulcanite experience was a little too musty. His saloons were very dirty; but my reason for an abrupt departure was an invitation to "step down cellar and see his three-horse power vulcanizer at work." If there isn't a blow-up from the said three-horse power, some fine morning, I am much mistaken.

I next called on an out and out "maker of dental fixtures, carved in the latest style, from the sea-horse tusk"—so ran the sign. I went up stairs, and found the venerable "Dentista" busy at work, fitting in one of the most stylish sea-horse fixtures I ever saw. So stylish and full of life was it still, though the parent animal was probably sleeping on the banks of the Nile, that it kept bouncing around, and finally landed on the floor, accompanied by an imprecation and some blood. I promised to see him again and went home.

A partial upper set lies before me, that I wish to send you, both as a specimen and a curiosity. The base is sea-horse tusk; the teeth human, screwed to the plate by platinum screws. It is made (so says the dentist) on "the suction principle." Its suction was sufficient to draw out a good price—300 francs for eight teeth. I will send you a few specimens, the first opportunity. I wish I could send you a moving German dental depot.

We are now using many English plain teeth, made by Ash and Sons, of London. They are superior to American plain teeth in shape, strength and color. Ash can not make gum teeth, and does not pretend to. We use a little of the English "pink rubber" for gums, with plain teeth. Ash has a workman from Abbey's making gold-foil. As to lathes, they make finer machines than can be bought in America; but they are deficient as to dental instruments, except forceps, and they *do* make most beautiful forceps.

Our practice here is among the better classes, and is very pleasant—no jewing down. Our prices for filling are from four to twenty gold dollars—artificial work, four dollars a tooth. Our competitors, of course, get any price they can. We can not use the mallet here, except on the English, on account of a marked tendency to periostitis. The Italians and Russians will not bear the mallet.

Absorption, with hemorrhage, comes constantly under our treatment. We manufacture a soap that completely cures it. It is composed of Tunis soap, chalk, chlorate of potash, honey, and arrowroot.

We prepare a metal for filling teeth, that in strength, color, and fusibility, challenges any other in use. I send you a small piece. It can be melted in the hand without pain. [This "metal" is something like Wood's; but we presume, from its appearance, that it contains mercury.]—Ed.

We have, for to-morrow, a most difficult case of cleft palate, the fissure being more than an inch wide.

Your Friend,
F. Slayton.

